# Fatal Delayed-Onset Bilateral Re-Expansion Pulmonary Edema After Pleural Drainage: A Case Report

**DOI:** 10.7759/cureus.95577

**Published:** 2025-10-28

**Authors:** Ayman Elsayed, Mohamed Shafei, John Hutchinson

**Affiliations:** 1 Respiratory Medicine, Sherwood Forest NHS Trust, King's Mill Hospital, Mansfield, GBR; 2 Respiratory Medicine, Sherwood Forest NHS trust, King's Mill Hospital, Mansfield, GBR

**Keywords:** chest drain, delayed onset, induced capillary leak syndrome, left sided pleural effusion, pleural drainage, re-expansion pulmonary edema

## Abstract

We report a rare case of bilateral re-expansion pulmonary edema with delayed onset occurring 12-24 hours after pleural fluid drainage in a male patient in his eighties. Unlike the typical presentation, which occurs within 1-2 hours, this case involved bilateral lung involvement and a significantly delayed onset. This observation underscores the importance of close monitoring and heightened clinical vigilance following pleural drainage, particularly in elderly patients with multiple comorbidities. Our case highlights the importance of being aware of both common and uncommon complications associated with pleural drainage, underscoring the value of adhering to current clinical guidelines.

## Introduction

Re-expansion pulmonary edema (REPE) is a rare but potentially life-threatening complication after thoracentesis, occurring in less than 1% of cases [1]. It occurs secondary to excessive intrapleural negative pressure, which leads to increased permeability and increased hydrostatic pressure in the pulmonary capillaries [2]. It was first reported in 1958 by Carlson et al. [3]. Several risk factors, including young age, diabetes, and smoking, may contribute to its development. It usually occurs following the rapid re-expansion of a chronically collapsed lung, especially after removal of more than 1,500 mL of pleural fluid in a single attempt [4]. Re-expansion after pneumothorax can also cause this complication, and it is generally more challenging to control the rate of re-expansion compared to drainage of pleural effusion [5]. It typically presents within the first hour of the procedure and is usually unilateral. This case contributes to the limited reports of delayed-onset and bilateral pattern of REPE that reinforces the importance of cautious drainage techniques.

## Case presentation

**Figure 1:**
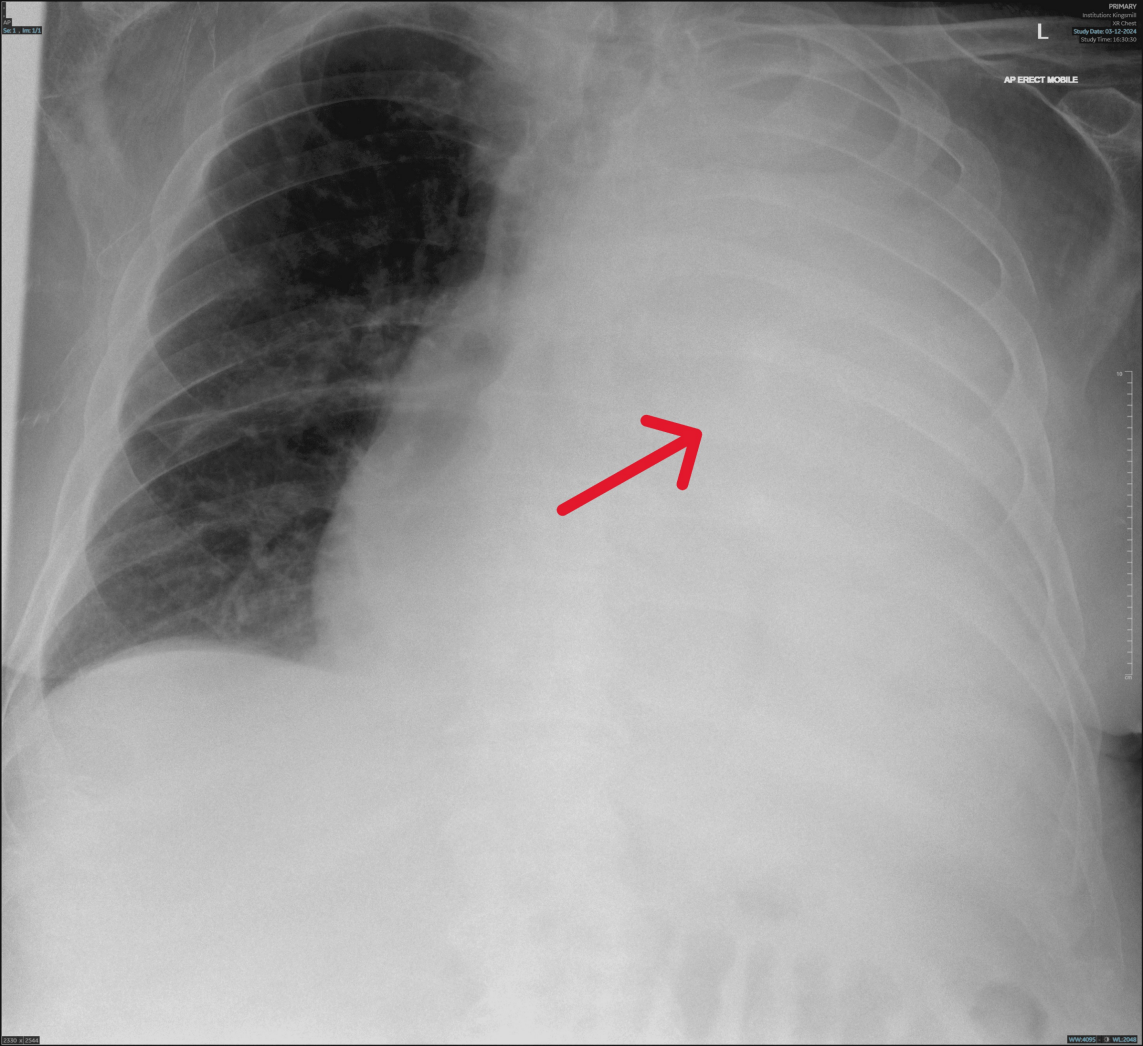
Chest X-ray (anteroposterior view) showing complete whiteout of the left hemithorax, indicative of massive pleural effusion. The right lung is clear.

An 85-year-old male with a past medical history of heart failure, atrial fibrillation, severe frailty, and type II diabetes mellitus presented to the hospital with new-onset shortness of breath and a new requirement for 28% oxygen via nasal cannula. Blood results showed high CRP levels at 98 mg/L (reference range: 0-5 mg/L), normal WBC count at 8.2x10^9^/L (reference range: 4-10x10^9^/L), and normal lactate level on the venous blood gas analysis. A chest X-ray revealed a massive left-sided pleural effusion (Figure 1).

The patient was referred to the respiratory team, and a decision was made to proceed with definitive management via intercostal chest drainage, given the significant size of the effusion and the low likelihood of requiring more invasive diagnostic testing due to his frailty. Following informed consent, a 12F chest drain was inserted without immediate complications, with a plan to drain fluid in a controlled manner as per BTS guidance [5].

**Figure 2:**
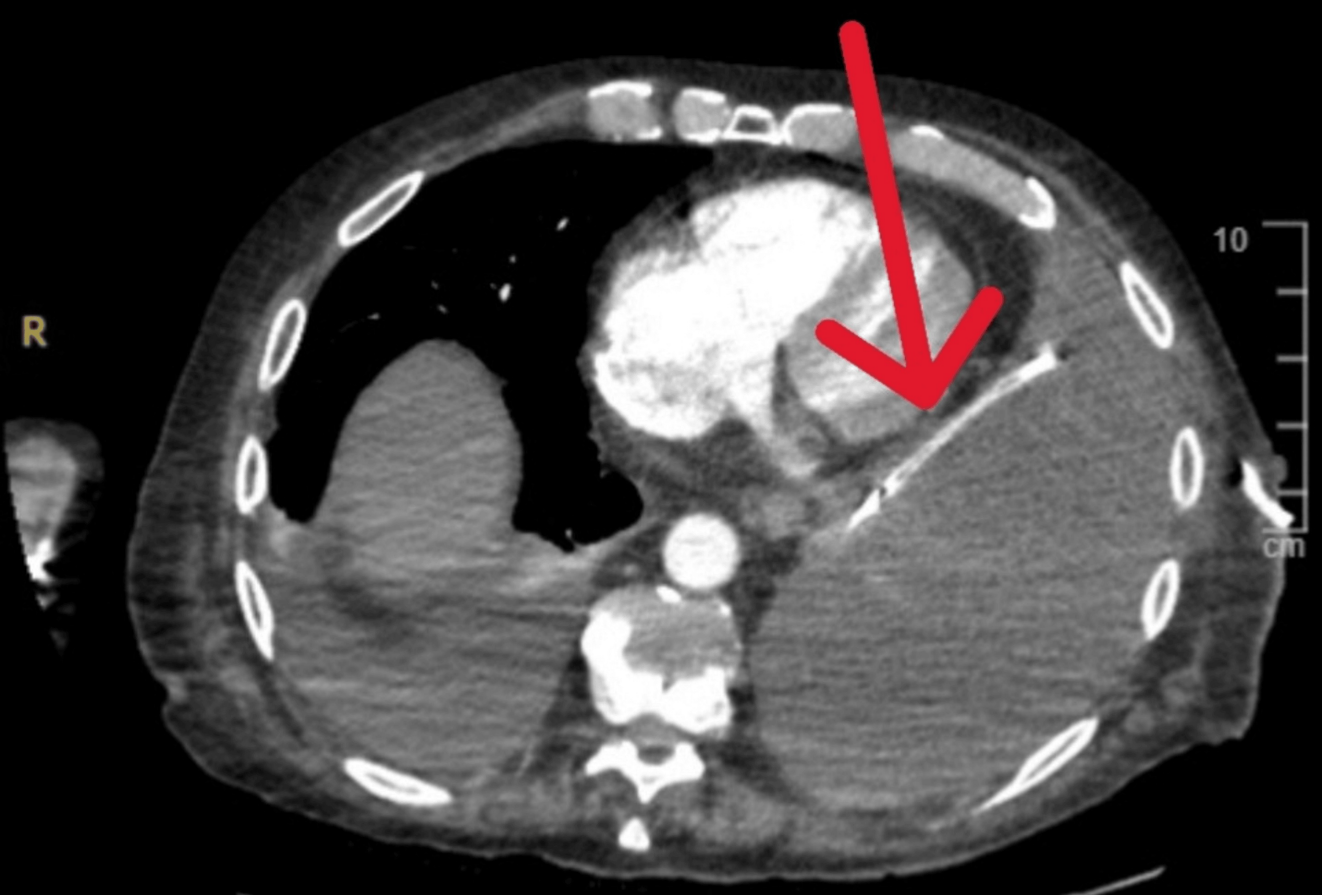
CT chest showing the left-sided effusion containing an intercostal drain with its tip medially.

A CT scan of the chest, abdomen, and pelvis with contrast was performed the same day after the procedure to investigate the etiology of the effusion, rule out malignancy, and confirm the placement of the chest drain. The scan confirmed the appropriate placement of the chest drain and showed no evidence of malignancy (Figure 2).

A total amount of 3,000 mL of pleural fluid was drained over 24 hours, and a sample was sent for cytology, lactate dehydrogenase, protein, WBC, and tuberculosis (TB) culture. The results were suggestive of an exudative nature of the sample, as the fluid protein was 36 g/L (exudate if > 35 g/L), with negative TB culture and no malignant cells.

**Figure 3:**
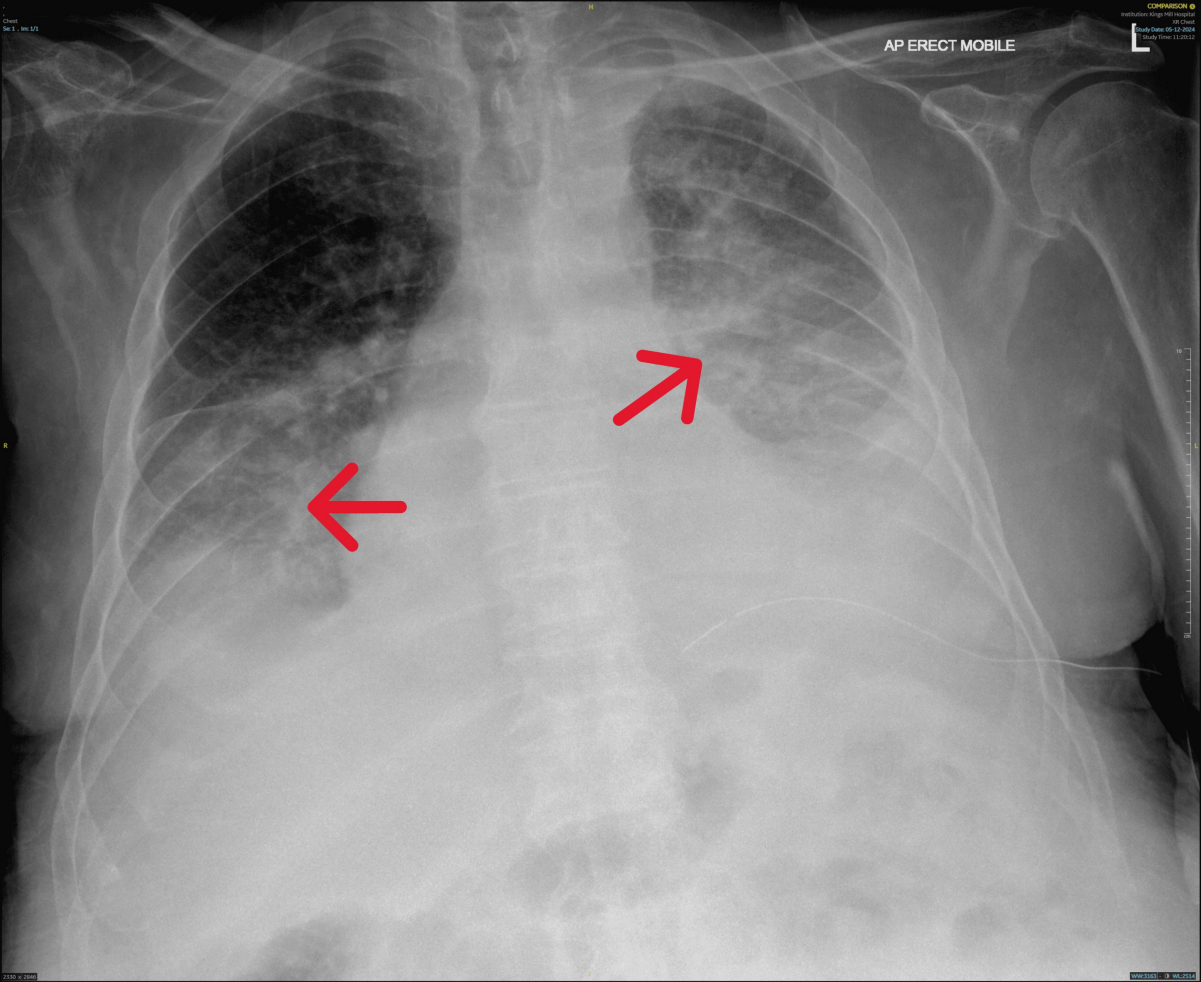
Chest X-ray showing bilateral patchy alveolar opacities predominantly in the dependent zones consistent with pulmonary edema, without cardiomegaly or pleural effusion recurrence.

Despite an initial improvement, approximately 12-24 hours post-drain insertion, the patient started to experience a sudden drop in oxygen saturation, accompanied by tachypnea and delirium. A repeat chest X-ray showed new bilateral pulmonary infiltrates consistent with pulmonary edema (Figure 3), which were more prominent in the left lung. Supportive treatment was initiated, including oxygen supplementation at 60% via a venturi mask, intravenous antibiotics (co-amoxiclav), and intravenous diuretics (furosemide) [4]. Due to the patient's significant comorbidities and an element of delirium, a higher level of care, including invasive or non-invasive respiratory support, was deemed inappropriate.

Despite active management, the patient's condition continued to deteriorate. After a multidisciplinary discussion and consultation with the patient’s family, a decision was made to start end-of-life care, prioritizing comfort and dignity. The patient died a few days later.

## Discussion

This case represents a rare presentation of bilateral REPE following pleural fluid drainage [6]. Classic REPE typically occurs within the first hour of drainage and is usually confined to the affected lung. In contrast, our patient developed symptoms much later, 12- 24 hours post-procedure, and demonstrated bilateral pulmonary involvement.

Risk factors for REPE include chronic lung collapse, younger age [7], rapid re-expansion, and high negative pleural pressures. Although this patient was elderly and underwent controlled fluid drainage, his advanced age, underlying frailty, and other comorbidities may have contributed to the poor prognosis.

The bilateral nature of the edema, involving the contralateral lung that had not undergone drainage, is particularly unusual and has been reported only rarely. The pathophysiology may involve systemic inflammatory mediators or capillary leak triggered by lung re-expansion [2].

The pulmonary interstitium is the connective tissue space bordered by the visceral pleura and situated between the alveolar epithelium and the capillary endothelium. It plays a critical role as a barrier regulating fluid movement between alveoli and capillaries. Changes in intrapleural pressure, particularly during rapid lung re-expansion after evacuation of large pneumothoraces or effusions, have significant effects on this space.

Rapid re-inflation of a collapsed lung can result in pressure-related mechanical stress and microvascular injury to pulmonary blood vessels, increasing their permeability. This mechanical injury is compounded by a sudden reversal of hypoxic pulmonary vasoconstriction, which acts as a pro-inflammatory trigger, generating oxidative stress and promoting the release of inflammatory mediators. These processes contribute to increased capillary leak and protein-rich edema formation.

Furthermore, the creation of significantly negative intrapleural pressures during the removal of large volumes of air or fluid lowers interstitial pressure, thereby increasing the gradient for fluid movement across the alveolar-capillary barrier. As a result, fluid accumulates within the lung parenchyma, producing the clinical manifestations of REPE [8].

## Conclusions

Bilateral REPE is a rare and severe complication of pleural drainage. This case highlights the need for continued vigilance beyond the typical REPE time window, especially in elderly or comorbid patients. Clinicians should consider delayed and bilateral REPE in the differential diagnosis when patients deteriorate after thoracic interventions.
